# Stress and Growth in Cancer: Mechanisms and Psychotherapeutic Interventions to Facilitate a Constructive Balance

**DOI:** 10.3389/fpsyg.2019.00177

**Published:** 2019-02-04

**Authors:** Cristian Ochoa Arnedo, Nuria Sánchez, Enric C. Sumalla, Anna Casellas-Grau

**Affiliations:** ^1^Psycho-oncology Unit, Institut Català d’Oncologia, L’Hospitalet de Llobregat, Barcelona, Spain; ^2^Institut d’Investigació Biomèdica de Bellvitge, Barcelona, Spain; ^3^Clinical Psychology and Psychobiology Department, Universitat de Barcelona, Barcelona, Spain; ^4^Hospital Clínic de Barcelona, Barcelona, Spain

**Keywords:** cancer, post-traumatic growth, post-traumatic stress, oncology, vicarious growth, secondary growth, psychotherapy

## Abstract

Post-traumatic stress and growth are common responses to adverse life events such as cancer. In this article, we establish how cancer becomes a “fertile land” for the emergence of stress and growth responses and analyze the main mechanisms involved. Stress-growth responses on adjusting to cancer is potentially determined by factors like the phase of the illness (e.g., initial phases vs. period of survivorship), patient’s coping strategies, meaning-making, and relationships with significant others. We also review the mechanisms of constructive and adaptative stress-growth balances in cancer to study the predictors, interrelated associations, triggering mechanisms, long-term results, and specific trajectories of these two responses to cancer. Finally, we update the evidence on the role of these stress-growth associations in psychologically adjusting to cancer. Together with this evidence, we summarize preliminary results regarding the efficacy of psychotherapeutic interventions that aim to facilitate a constructive psychological balance between stress and growth in cancer patients. Recommendations for future research and gaps in knowledge on stress-growth processes in this illness are also highlighted. Researchers are encouraged to design and use psychotherapeutic interventions according to the dynamic and changeable patients’ sources of stress and growth along the illness. Relevant insights are proposed to understand the inconsistency of stress-growth literature and to promote psychotherapeutic interventions to facilitate a constructive balance between these key responses in cancer.

## Introduction

Three stages can be identified in the history of research on psychological responses to adverse/traumatic situations such as cancer (Vázquez et al., 2014). The first stage, which goes from 1980 to the early 1990s, was lead by the trauma definition in DSM-III (American Psychiatric Association, 1980) in relation to vulnerability to stressors. During this stage, most studies were focused on the negative effects of trauma (Bonanno et al., 2010). The second stage took place during the mid-1980s, when it was found that serious life events do not necessarily cause mental disorders. For example, it was reported that despite significant national differences being largely unexplained, most participants (35–65%) showed being resilient when confronted with adverse events (Bonanno et al., 2011). We are currently in the third stage of research that focuses on positive aspects and growth resulting from traumatic experiences. The culmination of these changes is reflected in the profound transformation of the psychopathological conceptualization of trauma response in the fifth version of the “Diagnostic and Statistical Manual of Mental Disorders” (2013). In DSM-V, the new diagnostic category “Trauma and Stress-Related Disorders,” that includes both “post-traumatic stress disorder” (PTSD) and “adaptive disorder” (AD), redefines the concept of a traumatic event in a more restrictive manner. Thus, the new definition emphasizes that a traumatic event must refer directly to an exposure to a near–death experience, serious injury or sexual violence.

Cancer diagnosis, first recorded as a trauma in DSM-IV (American Psychiatric Association, 1994) is no longer considered a traumatic event. Currently, a medical event has to be urgent and catastrophic before it can be considered traumatic. Thus, existing therapies in oncology that improve prognosis and considerably increase survival rates make it difficult to define cancer diagnosis as a trauma (Brewin et al., 2009). Cancer is now redefined as a powerful stressor, but it does not have the potential to generate PTSD, except in very specific cases. The cancer patient’s own symptomatology, such as hyperactivation, avoidance and intrusion that were previously linked to PTSD, is now reconsidered under other diagnoses such as AD, generalized anxiety or somatized stress, with PTSD relegated to the background (Kangas, 2013). Moreover, the definition of trauma in DSM-IV (1994) included the type of response generated by the adverse event, requiring an emotional reaction of horror and intense fear for an event to be considered traumatic. In the new DSM-V (American Psychiatric Association, 2013), the subjective response of the patient is explicitly denied as a defining element of trauma, the objective characteristics of the event being the central criteria in understanding the trauma. Thus, the frequently catastrophic emotional experience in a patient diagnosed with cancer is no longer a defining characteristic of trauma (Pai et al., 2017).

The reformulation of criterion A in DSM-V (2013) could have important repercussions in the field of psycho-oncology in two different ways. First, it questions much of the theoretical apparatus from which the response to cancer diagnosis is addressed. According to Janoff-Bulman (1992), traumatic experience is characterized by the destruction or alteration of a whole series of basic beliefs about oneself, the world and others that allow the subject to generate a sense of security and meaning around their existence. Cancer diagnosis as a trauma, by affecting these basic beliefs, could generate feelings of insecurity and fear of the future, hinder interpersonal relationships or question one’s own value as a person, among other responses. However, if the cancer is stripped of its traumatic characteristics to become a simple stressor or adverse event, as posited by the new criterion A, the response associated with the trauma should be reconsidered under the adaptative response parameters. Second, all the reflections and practices focusing on the experience of cancer as a post-traumatic response (Rustad et al., 2012) should be reconsidered and reformulated as AD, as indicated by Kangas (2013), including stress and growth experiences as a common and clear moderator of psychological adaptation in cancer.

In this paper, we review the evidence to clarify and understand stress/growth responses in psychological adaptation during and after cancer treatment. First, we will perform a contextual analysis of cancer as a stressor with common factors that trigger trauma and growth. Second, we will review the mechanisms of constructive and adaptative stress-growth balances in cancer (cancer process, coping, meaning-making and relational syntony). Finally, we will detail growing evidence regarding psychotherapeutic interventions that facilate constructive stress-growth balances in cancer patients.

## Cancer as a Stressor and Common *Fertile Land* for Trauma and Growth: a Contextual Analysis

Within popular, mostly Western, culture, responses to adverse events such as cancer are increasingly being simplified into “being positive” or “being negative.” The scientific literature has contributed to this simplification, attributing positive responses to “growth” and negative ones to “stress-trauma.” The belief that traumatic and growth responses are independent and opposite to one another is a common error when interpreting outcome in diverse cancer studies. In this section, we will review the elements of cancer that help better understand the common and different bases of stress and growth within the same framework of human experience of the disease (Joseph and Linley, 2006).

In order to show a clear overview and analysis of stress-growth processes we decided to use a dichotomic conceptualization of these two extreme responses, to dilucidate their role in psychological cancer adaptation. However, numerous cancer survivors are resistant to or resilient against cancer related stress. We have deliberately decided not include resilience responses in cancer in part for the controversy over its meaning and overlap with growth processes. Many studies on growth have equated posttraumatic growth with resilience or considered growth a superior psychological functioning (Sumalla et al., 2009). In contrast, other authors suggested PTG and resilience should be viewed as two independent constructs. Moreover they argued that it is very unlikely for resilient persons to perform the meaning-making narratives characteristics of growth processess (Westphal and Bonanno, 2007).

### Same Origin of Stress and Growth Responses: Perception of Threat/Vulnerability

The processes of post-traumatic stress and growth in cancer, as well as in other extreme situations, have a common basis: the threat to one’s physical or psychological integrity (identity). Disease severity does not show clear associations with post-traumatic growth (PTG), however, the relationship between stress and growth begins in the high subjective perception of threat and vulnerability, which, in addition to an intense emotional reaction to the severity of the event, is narrowly associated with stress and growth in cancer patients (Cordova et al., 2001). The degree of the threat and challenge to one’s previous identity (basic beliefs about oneself, others and the world) may affect the subsequent responses of stress and growth in one’s search of a new balance and adjustment after the illness (Janoff-Bulman, 1992).

The relationship between stress and growth is clearly explained through the theory on the organismic valuing of adaptation to threatening events (Joseph and Linley, 2006). In their theory, the authors explain how both the emotional distress (e.g., post-traumatic stress) and PTG could be integrated into the same structure of human experience. They distinguished two main procedures in adjusting to threatening events: assimilation and accommodation. Assimilation focuses on the stressful event management and the human willing to integrate this event into one’s basic beliefs, in order to keep them from changing. This occurs in the time around the traumatic event (peri-traumatic) and generates appraisal meaning mechanisms that aim to control or regulate intense emotional reactions after the event. The maintenance of post-traumatic stress is a global indicator of difficulties or dysfunction in the assimilation process, indicating the need for cognitive elaboration of the information challenged by the traumatic event (e.g., mortality). Often, to elaborate this information, people must make changes to their basic beliefs, which leads to the accommodation process that focuses on creating new vital meanings. Accommodation is composed of changes in identity that one performs when incorporating their understanding of an extreme experience. This may either generate meanings of chaos, absurdity or terror, resulting in trauma (negative vital changes), or, on the contrary, generate searches, deepening and endowing new vital meanings that result in growth (positive life changes). However, in most cases, a combination of both responses occurs together: trauma and growth (Sumalla et al., 2009). The centrality that the event has on one’s life is a triggering factor for the initiation of these assimiliation and accommodation processes (Reiland and Brendan Clark, 2017; Wamser-Nanney et al., 2018). As such, the most central an event is in one’s life, the most these processes are ought to emerge (Reiland and Brendan Clark, 2017; Wamser-Nanney et al., 2018). The next section and Table 1 clearly state cancer as an example of how these processes work in the aftermath of trauma.

**Table 1:** Cancer characteristics and stress-growth responses.

Cancer characteristics/responses	Stress-Trauma response (negative changes)	Growth response (positive changes)
Internal source (body)	Body hypervigilance and alert (health anxiety).	Self-care and adoption of healthy lifestyles.
Future perception of threat	Feeling of limited future.	Change of life priorities and greater appreciation for life.
Permanent, undefined threat (complexity)	Ongoing threat, existential trouble and *Sword of Damocles* syndrome.	Maintenance of structural identity changes.
Perceived control	Guilt and shame.	Responsibility and involvement in the process (adherence to treatment).
Invalidating sequels	Incomprehension, loneliness, alienation.	Need for others, gratitude, closeness and openness.

### Characteristics of Cancer as a Stressor in the Common Basis of Stress and Growth Responses

Current and past debates about cancer as a potential traumatic event have focused on the extent to how well cancer fits in a biomedical model of trauma based on acute stressors. Several authors (Smith et al., 1999; Kangas et al., 2002; Mehnert and Koch, 2007; Sumalla et al., 2009) have emphasized the distinctions between cancer diagnosis and treatment and other acute adverse events. The differences between cancer and other stressors have focused on the traumatic response, but not on post-traumatic growth. In Table 1, we exemplify how the characteristics of cancer could promote the usual stress-trauma and growth responses in a common framework of human experience. For example, the internal source of cancer (appears in our body) partly explains hypervigilance and health anxiety (stress response), which trigger self-care and the adoption of healthy lifestyles (growth). Psycho-oncological treatments and natural adaptation processes promote a balanced response of a salutogenic consciousness of one’s body (alert) to promote better self-care, without seeing the body in constant danger.

## Mechanisms of Constructive and Adaptative Stress-Growth Balances in Cancer

The paradoxical coexistence of posttraumatic stress and PTG in cancer is one of the most interesting areas with very few substantial clinical investigations (Sumalla et al., 2009; Ochoa et al., 2017). The first set of studies in this field explored the association between stress and growth, finding significant and positive associations between them (Bower et al., 2005; Kilmer et al., 2009; Xu and Liao, 2011; Lowe et al., 2013; Liu et al., 2018). The interpretation of this association remains unclear. For some authors, this association indicates that the complex combination of being conscious and having both negative and positive experiences may represent evidence of real growth (Butler, 2007). However, other authors doubt the adaptive value of growth and consider its illusory face (Zoellner and Maercker, 2006). The second set of studies found no significant relationship between the psychological responses of stress and growth (Cordova et al., 2001). This has been interpreted as an indicator of the need to view stress and growth as two separate processes (Salsman et al., 2009; Shakespeare-Finch and Lurie-Beck, 2014). However, the third set of studies and reviews showed a more negative correlation between stress and growth (Frazier et al., 2001; Sawyer et al., 2010; Hall et al., 2015). In general, these third set of studies results highlighted the adaptive value of growth as a buffering and direct effect in reducing stress and discomfort (Wang et al., 2014).

All these results demonstrate that a clearer conception on the combination of stress and growth responses and the role they play in the positive adjustment to cancer are needed. Zoellner and Maercker (2006) introduced the distinction between the constructive and illusory aspects of PTG, which other authors have associated with positive real or illusory changes (Sumalla et al., 2009). Constructive growth describes the functional aspects of positive changes, while illusory growth defines dysfunctional or self-deceptive growth. The model assumes that the two aspects of PTG can simultaneously occur and are likely to involve different paths and mechanisms. Constructive growth is more probable to produce positive adaptation at long term, while illusory PTG offers short-term relief that is likely to decrease over time (Zoellner et al., 2008).

To explore the different adaptative significance of this paradoxical coexistence and explain the relevant mechanisms and factors associated with the constructive and adaptative stress-growth balances in cancer, we analyzed the role of time and the càncer process, coping and emotional regulation, continuity and coherence of meaning-making, and relational stress-growth syntony.

### Time and the Cancer Process

Studies focusing on how stress and growth interact during the cancer process report contradictory outcomes. The different adaptive and dynamic meanings of the combination of stress and growth during the entire cancer process can help us understand and clarify their role in the process of facing and adapting to cancer.

The coexistence of stress and growth in the initial stages of cancer (diagnosis and treatment) has been linked to its illusory nature, seen as cognitive avoidance and a short-term palliative coping strategy that is lost over time (Sumalla et al., 2009). A similar palliative or buffering effect has been found in predictive studies. Although these studies found that growth is more likely to produce long-term positive psychological effects and less subsequent distress (Carver and Antoni, 2004), others observed that growth predicted either higher distress or that it was unrelated to future distress (Tomich and Helgeson, 2004; Bower et al., 2005). In fact, a longitudinal study by Lechner et al. (2006) showed that current growth (measured as a search for benefits) did not indicate reduced current or future stress, but the increase in growth over time was associated with a decrease in stress. In other words, it is the dynamic process of increasing growth throughout the illness (learning) that predicts the decrease in stress and not the early willingness to change when the threat is still current, this growth having a more defensive and illusory role.

Wang et al. (2017) recently reported some results that could explain the controversial results of Lechner et al. (2006) involving a common moderator between the stress and growth responses in cancer, perceived vulnerability. The authors referred to vulnerability as negative changes in the perception of physical vulnerability, which included the fear of cancer recurrence, concerns about the side effects of cancer treatment concerns, and feeling the world as a more unsafe place. In the study, the only relationship between PTG and distress might have been shadowed by the positive association between stress-growth and vulnerability. Thus, extremely narrowing the focus on growth or stress can channel to a misleading conclusion about adjusting to the illness (Bellizzi et al., 2007; Park and Blank, 2012). Wang et al. (2017) showed that growth independently predicted lower distress or stress over time after having controlled for vulnerability, confirming the adaptiveness of growth to reduce or buffer against stress in cancer.

Based on the abovementioned data, psychological treatments in cancer may need to be tailored according to stress-growth and time (Ochoa et al., 2017). In the initial phases during diagnosis and primary cancer treatment, vulnerability is linked to the need to increase emotional awareness and regulation with psychoeducation or stress management. In these stages, stress management and psychoeducational therapies focused on understanding and reducing the threat of the initial stressors (surgical intervention, chemotherapy and radiotherapy) would be suitable, as patients are in the assimiliation process. After primary cancer treatment, it would be more relevant to provide therapies facilitating growth such as meaning-making therapies, as patients begin to accommodate their experience and become open to considering vital changes.

### Coping and Emotional Regulation

The numerous studies that link coping with stress and growth in cancer reveal the importance of coping styles in cancer-related stress and growth. These responses are triggered by the self-assessment of cancer as a potential traumatic or threatening stressor (Cordova et al., 2007; Andrykowski et al., 2015), generating automatic rumination that increases stress. Depending on the coping strategies used, this automatic rumination can increase stress (trauma) or elicit deliberate rumination that leads to a re-elaboration of the experience into positive life changes (growth).

Post-traumatic stress symptoms and PTSD have been linked to non-adjusted coping strategies (Jacobsen et al., 2002), such as anxious worrying (Pérez et al., 2014), cognitive avoidance, helplessness, fatalism (Pérez et al., 2014), self-blame, denial, and behavioral disengagement (Richardson et al., 2016; Langford et al., 2017). Furthermore, PTG has been directly linked to the patient’s active coping strategy (Bellizzi and Blank, 2006; Lelorain et al., 2012; Svetina and Nastran, 2012; Tong et al., 2012; Danhauer et al., 2013) problem solving (Widows et al., 2005), positive reappraisal (Sears et al., 2003; Carver and Antoni, 2004; Urcuyo et al., 2005; Widows et al., 2005; Lechner et al., 2006), religious coping (Urcuyo et al., 2005; Jim et al., 2006; Lechner et al., 2006; Gall et al., 2011), and acceptance (Urcuyo et al., 2005).

We therefore conclude that PTG could be linked to active coping, focusing on both the problem and the emotion, while PTSS/PTSD could be associated with dysfunctional avoidance coping. There are few studies exploring the role of coping as a mechanism to elucidate constructive/adaptive or illusory stress-growth balances in cancer. Available data show that during the initial assimilation process of adjustment, when coping strategies focus on the emotion (stress), the coexistence of growth is usually temporary and does not imply a change in one’s belief system (Sumalla et al., 2009). This process could be related to an illusory PTG, which is defensive and temporarily produces a positive emotional state and reduces stress. However, these emotional states are not maintained over time. Accommodation and constructive growth are more likely to maintain meaning-based coping processes, involving an active search for meaning that would lead to more general, deep and stable changes in one’s perception about oneself, others and the world. Accommodation is fundamental in constructive PTG and is associated with active coping that includes positive reappraisal, acceptance and behavioral changes sustained over time (Pat-Horenczyk et al., 2015). Furthermore, the flexibility of coping could also be important in developing constructive PTG (Pat-Horenczyk et al., 2016).

Some longitudinal data dispute these general processes of assimilation and accommodation. For example, there are studies showing that those who use coping styles that facilitate emotional expression and communication in stressful events during the early moments of diagnosis and treatment show better growth and less stress later (Manne et al., 2004). Therefore, patients who show high and rapid emotional re-elaboration, together with a meaning-making coping style, tend to report high growth scores that reflect an early and constructive growth as a result of deep pre-conception and pre-cancer beliefs, which are praised and prioritized after the disease. A sentence that exemplifies these situations is: “I always thought that I had to prioritize the family and now it has become a pressing vital change to do because of the disease.” Moreover, Pat-Horenczyk et al. (2015) emphasized the importance of the temporal dimension. PTG develops during a process of dynamic and changing adjustment, with individuals experiencing illusory and constructive PTG as subsequent steps in the adjustment process.

### Giving Meaning to the Experience: Continuity and Coherence of Meaning-Making Narratives

Stress and growth are responses that are closely linked to events that psychologically challenge one’s basic beliefs. That is, they question the basic psychological framework of understanding and the meaning to life (Janoff-Bulman, 1992). Finding meaning in life is one of the primary motivations of a human being. Meaning-making is a process by which people create, understand or give meaning to events in life, relationships and oneself. It is considered essential in adjusting to stressful situations (Gillies and Neimeyer, 2006). In her review of the studies on meaning-making, Park (2010) synthesized the concept of meaning as “meaning connects things.” Cancer survivors, attempting to bring coherence and continuity to their life experience, may look forward to integrating and giving answers to the traits of their life that are challenged by their illness. Park (2010) distinguished two components in the process of meaning-making: (a) the meaning-making process, which includes coping efforts to understand the stressor (appraised meaning) and include it in the individual’s global belief system and (b) the products of this process, the meaning made, which are the final results of the search for meaning. The most important within these meanings would be (1) the perception of PTG, (2) a sense of meaning of the deeper life, and (3) the restoration or reduction of the inconsistency of just-world beliefs.

Empirical studies on people with cancer show inconsistent results regarding the role of meaning-making in the psychological adjustment to cancer. In some papers, the search for meaning has been related to better adjustment and quality of life (Davis et al., 1998; Sears et al., 2003; Bower et al., 2005). However, in other studies, searching for meaning has been associated with higher levels of stress and a lack of adjustment. Other studies show that meaning-making moderates the effects that intrusive thoughts (Park et al., 2010) and social and physical functioning (Jim and Andersen, 2007) have on stress. Some researchers claim that people who do not try to find a meaning are equal or even better than those who do (e.g., Bonanno et al., 2005). Some theorists point out that meaning-making only favors psychological adaptation and reduces stress when a meaning is found (Segerstrom et al., 2003). While meaning-making does not result in any change that reduces the inconsistency with global meaning, it does positively correlate with stress. If results are satisfactory (meaning made), the need to continue searching for meaning ends and, so, the stress ends. Meaning-making may not be adaptive if it is highly intensive and maintained over a long period without any result (Joseph et al., 2005). In conclusion, although the search for new meaning is the basis of PTG for most patients, meaning-making could be more constructive and adaptive if it solves and canalizes unproductive ruminations about, for example, the fear of cancer recurrence or other threats (cognitive post-traumatic stress symptoms) (Ochoa et al., 2017).

It is widely accepted in clinical settings that the way to reduce post-traumatic stress and improve PTG in meaning-making interventions is by creating sustainable continuity and coherence with pre-cancer identity narratives, now enriched and integrated with information on threat and mortality (Ochoa and Casellas-Grau, 2015). New approaches, such as positive psychotherapy, propose new strategies to work with in the search for meaning in cancer. In positive psychotherapy, the construction of continuity and biographical coherence is not only carried out by working with traumatic memories, but through the recovery of positive autobiographical memories and establishing patterns of personal fulfillment (Serrano et al., 2004; Ochoa et al., 2010). These patterns link relevant aspects from the past, present and future (Guidelines of Personal Realization), reducing stress and favoring PTG in cancer (Ochoa et al., 2010; Vázquez et al., 2014).

Some authors summarized the role of the search for meaning in stress/growth responses in a comprehensive way (Bauer and McAdams, 2004). This search for meaning in situations that promote stress/growth may be better understood as a process during which a narrative that explains how the person has been positively transformed by the traumatic event is constructed and then integrated into an identity-defining life story. For them Bauer and McAdams (2004) the life story about suffering should not be “just one piece of the complex puzzle of posttraumatic growth (…) but rather as the fundamental frame that holds the entire puzzle together.”

### Relational Stress-Growth Syntony

One of the foundations for our psychological life is the desire for interpersonal relationships. Then, personal stress and growth responses should be regarded as being linked to the deterioration and optimization of interpersonal relationships. To understand stress-growth responses, as well as their triggering mechanisms and their balancing adaptive value during the cancer process, it is necessary to know the relational impact of a patient’s significant others. Cancer may constitute a stress/growth experience for both patients and their significant others with whom share the experience of the illness (Ochoa et al., 2013). An increasing amount of research have reported the presence of distress or stress among cancer patients’ significant others, especially partners (Hodges et al., 2005) and the parents of children or teenagers suffering from cancer (Landolt et al., 2003; Ozono et al., 2007). Moreover, patients’ significant others and caregivers have also been reported to experience growth and positive changes (Weiss, 2004a; Cadell, 2007; Zwahlen et al., 2010; Moore et al., 2011), indicating that the effects of trauma and growth in the aftermath of disease are not exclusive from survivors, as they also have relevant effects on those accompanying or helping them, or even those who simply witness their suffering. The relevant factors in relational stress-growth responses between cancer survivors and their significant others are: independent or shared relational stress-growth responses, the direction of this influence, and corroboration/congruence between them to discriminate between constructive and adaptive relational combinations. In Table 2, we have adapted and extended the contents of our first review (Ochoa et al., 2013) on how cancer could become a secondary traumatic stress/growth (independent of the trauma or growth in the cancer patient) or a vicarious stress/growth (transmitted and related to the trauma and growth in the cancer patient) event in the significant others. Moreover, our adaptation helps to elucidate some key points in relational stress/growth responses between cancer survivors and their significant others to discriminate between constructive stress/growth combinations.

**Table 2:** Distinction between secondary and vicarious stress/growth responses.

Cancer survivors/significant others	Secondary posttraumatic stress/growth	Vicarious posttraumatic stress/growth
Does the significant other’s stress/growth result in definitive changes in their life?	This is a primary stress/growth and is more significant than for the survivor.	The degree of stress/growth is the same as for the survivor.
Who initiates and generates the stress/growth responses?	They are initiated by the significant other and how the fact challenges their identity.	They are initiated by the survivor’s PTG, which predicts, drives or affects the significant other’s PTG.
Are there asymmetrical processes of stress/growth transmission?	Stress and growth are parallel or symmetrical. The significant other’s stress and growth may be greater than the survivor’s.	Asymmetrical. From the survivor to the significant other, through observational, relational, modeling, transmission or imitation learning.
Are the sources and dimensions of stress/growth similar or different? Is there harmony and synchrony within the answers?	No. As they are independent processes, stress and growth may arise for different reasons and in non-shared dimensions.	Yes. Stress/growth arises from similar sources and dimensions. There is harmony and synchrony in the answers.
Importance of relational and family variables	They are not important. Stress/growth responses are autonomous and independent, and so are essentially an intrapersonal process.	They are the basis of the significant other’s stress/growth. These variables predict and mediate the changes in both the significant other and the survivor, as growth is an essentially interpersonal process.

Overall, Ochoa et al. (2013) reported significant correlations between the stress/growth responses of cancer survivors and their partners. However, a more detailed analysis revealed that the mechanism of stress/growth “transmission” to the partner of a cancer patient differed depending on gender. Growth in men who had a female partner with cancer was lower than that in the patient but was predicted by and depended more on the growth of their female partner, suggesting vicarious learning or transmission (Pakenham, 2005; Ackroyd et al., 2011). By contrast, stress/growth in women who had a male partner with cancer was similar to or greater than that in the patient, which could emerge in different dimensions to that of their spouse (Thornton and Perez, 2006; Ruf et al., 2009; Zwahlen et al., 2010). For example, Ruf et al. (2009) found that, on the one hand, women placed greater emphasis on the improvement of their marital relationship (reporting increased intimacy and communication), while, in the other hand, male patients focused the description of positive changes in their family relationships and friendships, rather than in their marital relationship. Other studies have reinforced this gender effect, especially in growth responses. Female cancer survivors can transmit more growth to their partners and female partners of male cancer survivors can show more growth than their partners. Moreover, growth in female breast cancer survivors can be induced by other women with breast cancer more than by their own husbands (Weiss, 2004a,b).

Studies on the predictors of stress/growth responses in couples have only found two common shared factors: positive reframing and age (Ochoa et al., 2013). Age may favor a shared stress/growth response probably because, for both parties, cancer may be a very disruptive unexpected event in young people (trauma) and because younger people are prone to have a greater willingness and capacity for change (growth). Positive reframing seems to be a mediating coping style in common stress/growth responses in partners. The focus of positive reframing on what was achieved instead of what was not achieved in cancer could be related to positive peri-traumatic meaning-making being closely linked to the PTG accommodation process.

Similar results have been reported for other close relationships, like the parents of children or teenagers suffering from cancer. About the 50% of these parents report stress symptoms, and 20–25% of them meet the PTSD diagnostic criteria (Pelcovitz et al., 1996; Ozono et al., 2007). In a similar way, however, parents of children with cancer do also report positive psychological changes (Barakat et al., 2010; Hungerbuehler et al., 2011). It is not well understood how these stress/growth responses are transmitted or shared in parental relationships. However, there is more evidence about the traumatic effect (stress symptoms) that these experiences have on parents than there is for growth transmission. Indeed, parents (especially mothers) show a greater predisposition to report high post-traumatic stress scores than their child, despite it was this latter who suffered the disease. Further, mothers tend to show a higher prevalence of stress symptoms than adult survivors of cancer, what suggest the higher traumatic nature of the experience of having a child with cancer than the direct experience of the disease (Smith et al., 1999; Kissane et al., 2003). Studies examining longitudinal stress/growth processes in fathers, mothers, and children separately have reported interesting results. Hungerbuehler et al. (2011) reported that mothers experienced more psychological distress than fathers 1 month after the cancer diagnosis of their child, this distress being associated with higher levels of growth three years later in the mothers but not fathers. Again, it seems that synchronic emotional distress (and their expression) at diagnosis could promote growth over time. Since it is unclear whether growth in the mother is associated with that in the child or father, only speculative hypothesis about its vicarious or secondary nature can emerge. However, some studies suggest that mothers play an essential role in transmitting stress/growth responses to the child (Pelcovitz et al., 1996) because their responses appear to be higher and better predictors of stress/growth responses in children and fathers (Barakat et al., 2006; Hungerbuehler et al., 2011). To summarize, illness in a child or teenager is more likely to produce initial stress/trauma and future growth in mothers than in fathers.

Data on relational stress/growth responses in cancer have also been used to assess the real, adaptive and constructive nature of these responses in cancer. The transmission or corroboration of growth in the significant others of patients indicates “real, adaptative and constructive” relational syntony (Shakespeare-Finch and Enders, 2008; Sumalla et al., 2009; Moore et al., 2011; Ochoa et al., 2013). However, some studies show that memories about interpersonal growth are far from precise (Tennen and Affleck, 2009) and may be subject to bias. Furthermore, agreement in couples regarding growth does not ensure the presence of a real change (Kirkpatrick and Hazan, 1994). Couples may rewrite memories and show memory bias when recalling their shared history, highlighting positive aspects of their emotional life which had not been recounted before. However, this positive memory bias could have a constructive outcome. Positively remembered experiences have been shown to be a better predictor of well-being than the veracity or accuracy of these memories (Wirtz et al., 2003).

Some other important mediators of these real, adaptative or constructive stress/growth relational combinations have been found. For example, stress/growth concordance in couples (Ruf et al., 2009) is linked to higher mutual future growth than discordance in couples, which results in low satisfaction in their relationship and more separations. Other variables like flexibility, perceived family cohesion and quality of family relationships correlate with lower stress symptoms in teenagers with cancer (Pelcovitz et al., 1996) and predict more growth (Hungerbuehler et al., 2011). All these relational/family mechanisms sustain mutual vicarious learning and support, making cancer a “family shared seismic event” that may buffer against stress and promote relational growth (Ochoa et al., 2013).

### Personal Characteristics and the Stress-Growth Relationship

Personal charactesristics have also been explored as underlying mechanisms of the stress/growth relationship. A recent systematic review (Casellas-Grau et al., 2016) concluded that, in breast cancer, the age of patients had a distinctive role in triggering high levels of stress, but also, promoting posttraumatic growth. The explanation that sustains this paradoxical relationship is the perception of cancer as more disruptive and aggressive among younger women than in their older peers (Kangas et al., 2005; Ochoa et al., 2013; Sharp et al., 2018).

Future prospective research on the mechanisms of stress/growth among close relationships in cancer is required. Correlational data still prevail, making some interpretations speculative. However, available data suggest a clear effect of gender and/or role (mother vs. father). Female cancer survivors promote more vicarious stress/growth responses in their male partners, while female partners of male cancer patients or mothers of children or adolescents with cancer can show even greater stress/growth than the patients themselves. This indicates that for women, cancer in their significant others constitutes a secondary stress/growth process (in this case, the response being more independent from that of the patient) and for men, cancer in their significant others, trigger stress/growth processes that are more vicarious and dependent of their cancer survivors loved one. These stress/growth responses are constrained by important aspects linked to the synchronicity of their responses (Stanton et al., 2000). Likewise, modulating variables, such as positive reframing, or relational variables, like relational concordance or family cohesion, show greater capacity to generate mutual vicarious growth and could be important in the shared reduction of stress in cancer (Pelcovitz et al., 1996; Ruf et al., 2009; Ochoa et al., 2013). Also the marital status and the special and concrete social support provide by one’s partner has been a focus of exploration among the latest literature. Studies have found that this type of social support has a stress-absorbing function for patients in two ways: on the one hand, it buffers the negative effects of stress, especially in the first phases of the disease, and in the other hand, it promotes the emergence of positive emotions due to the closer and intimate relationships, deriving in stress reduction and growth facilitation (Salovey et al., 2000; Cohen, 2004; Kangas et al., 2005; Shand et al., 2018).

## Preliminary Evidence From Psychotherapeutic Interventions Facilitating Constructive Stress-Growth Balance in Cancer Patients

In this paper, we have shown how research on the psychological responses to traumatic or adverse events such as cancer has been changing to incorporate the combination of stress and growth responses in the adjustment process. Few (recent) studies have clarified and contextualized this combination of responses in such a complex disease as cancer. Moreover, few articles have proposed psychological interventions to achieve a constructive and adaptive balance of both responses (Pat-Horenczyk et al., 2016; Ochoa et al., 2017).

In a recent review of interventions aimed at facilitating growth (Roepke, 2014), the authors could not find good studies that used treatments facilitating growth. In fact, the first interventions that managed to promote growth in patients with cancer achieved it as a side effect, since they were designed to focus on and target stress management (Antoni et al., 2001, 2006; Bower and Segerstrom, 2004; McGregor et al., 2004; Penedo et al., 2006). In addition, these interventions are performed in the initial stages (diagnosis and treatment) of the disease when cancer stressors are present, using a measure of growth that is closely linked to seeking benefits during coping. These results confirm what was explained in the previous section. Soon after cancer diagnosis, stress and growth tend to occur together, reflecting a reaction to perceived vulnerability. Stress management in the initial phases of cancer could promote constructive and adaptative stress-growth, buffering against post-traumatic stress symptoms. However, what happens when significant stress and distress remain? Would an intervention facilitating PTG be more suitable in cancer survivors after primary cancer treatment than an intervention targeting stress management?

PTG facilitation is associated with the positive psychology scientific movement. A recent systematic review (Casellas-Grau et al., 2014), based on positive psychological interventions for breast cancer survivors, concluded that these interventions can result in an increase of PTG, well-being, meaning, quality of life, hope, optimism, happiness, benefit-finding, and, life satisfaction. Further, the most effective interventions are those performed with samples coming from hospitals, those which have an individual self-help style, and those that are longer (Bolier et al., 2013). However, none of these studies designed the intervention to understand stress and growth in cancer, with the mechanisms underlying these positive effects remaining unclear.

The first intervention that focused on facilitating PTG to reduce post-traumatic stress was Positive Psychotherapy in Cancer (PPC) (Ochoa et al., 2010; Ochoa and Casellas-Grau, 2015). The intervention was designed after having extensively reviewed the literature exploring trauma and growth processes in the aftermath of cancer (Sumalla et al., 2009). PPC integrates trauma and growth into the same framework of human experience to obtain a stress-growth balance. The effectiveness of PPC has been proved in pilot studies. It achieves greater reductions in emotional distress and post-traumatic stress and facilitates PTG compared to a waiting list group (Ochoa et al., 2017) and another cognitive behavioral stress management therapy that improves psychosocial adjustment (Antoni et al., 2001). In the pilot study, PPC was superior to this latter stress management therapy in reducing post-traumatic stress, emotional distress, and facilitating PTG at 3- and 12-month follow-ups (Ochoa, 2012). In studies with larger samples (Ochoa et al., 2017), the significant reduction of post-traumatic stress favored by PPC was related to an increase of PTG. Thus, as reported in other studies, PTG predicts better adaptation after the disease, showing better mental health and a better physical health subjective state (Helgeson et al., 2006; Sawyer et al., 2010). Further, there is an association between high levels of post-traumatic stress and a loss in the quality of life in cancer patients (Cordova et al., 1995) and this loss is lessened by experiencing PTG (Morrill et al., 2006). In conclusion, growth can be a therapeutic way to enhancing quality of life in survivors.

Another similar intervention (Pat-Horenczyk et al., 2015) studied the illusory and constructive aspects of PTG in a group of breast cancer survivors participating in a group intervention that aimed to build resilience. During a 6-month period, more than half of the participants reported PTG, and the intervention group reported a higher increase in coping and PTG than the control group. Moreover, participants in the intervention group reported more constructive growth (improved coping and increased PTG) and less illusory growth (increased PTG, but no differences in coping improvement) than those in the control group. This demonstrates the effectiveness of interventions that enhance coping and promote PTG. This intervention was specifically focused on promoting self-regulation strengths, cognitive restructuring, and active coping, while discouraging avoidance among breast cancer survivors.

To conclude, the few intervention studies that have been carried out to achieve a better stress-growth balance indicate the importance of the elements reviewed in this work. First, cancer is a stressor with common precursors for trauma and growth, especially perceived vulnerability. Psycho-oncological interventions focused on stress-growth responses need to facilitate growth in relation to vulnerability (emotional awareness and expression), as well as better self-care behavior, changing priorities and a need for others (openness and closeness). Second, psychological adaptation has changing sources of stress/growth during the cancer process that influence our targets and psycho-oncological interventions. We have detailed the most important mechanisms of constructive and adaptive stress-growth balances in cancer: the role of time and the cancer process, coping and emotional regulation, continuity and coherence of meaning-making narratives, and relational stress-growth syntony. Third, recent innovative psychological interventions have taken into account stress/growth procesess in cancer to facilitate a constructive balance. It should be noted that the interventions that facilitate PTG to reduce stress are more recommendable after cancer treatment. Moreover, interventions must promote assimilation first and then accommodation, prioritizing self-regulation, active coping and cognitive restructuring, while discouraging avoidance. Accommodation and growth are promoted by meaning-making and greater relational syntony with the significant others of cancer patients.

## Future Research

These conclusions lead us to encourage future research in developing and promoting the use of psychological interventions based on promoting growth, especially for those patients reporting higher levels of stress. We suggest investigating how to properly apply these psychological interventions, taking into account the changeable sources of stress-growth during the cancer process. Specifically, in the first phases of the disease, -the diagnosis and first oncologocical treatment-, it would be necessary to focus the intervention on the stress reduction through more directive psychoeducational therapies and emotional regulation interventions. However, future research should address whether the interventions that facilitate growth in these early stages can also reduce stress. To our knowledge, there are no current studies exploring this field. On the other hand, after having completed the primary cancer treatment, psychological treatment could be better focused on growth facilitation, where peritraumatic stress associated with threat and vulnerability around treatments are further away. In addition, as aforementioned, social support and relational growth are also a relevant focus of interest, given the positive synergic influence these have on cancer patient’s process of growth facilitation and stress reduction. For this reason, future research should not only focus on patient itself, but also on their significant others, feeding a positive retroalimentary circle between them.

## Author Contributions

COA was a clinical psychologist, an expert in psycho-oncology and the principal investigator. As the first author, he has been in charge of articulating and coordinating all the work of the rest of the authors. He has reviewed, adapted and drafted most of the contributions, with special emphasis on sections two, three and four. NS is a clinical psychologist expert in psycho-oncology. Her collaboration has been based on reviewing the role of coping and the meaning-making processes on stress and growth processes in cancer in section three. ES was a historian, clinical psychologist and anthropologist. His collaboration has focused on the history, conceptualization and phenomenology of cancer as a stressor that facilitates trauma and growth processes in cancer. He has collaborated mainly in the introduction and in the two tables that clarify these processes. AC-G was a PhD in psychology and an expert in positive psychology in cancer. His collaboration has focused on the review of interventions based on the positive psychology in section Preliminary Evidence From Psychotherapeutic Interventions Facilitating Constructive Stress-Growth Balance in Cancer Patients and has given methodological support to all authors in the bibliographic and critical search of the paper.

## Conflict of Interest Statement

The authors declare that the research was conducted in the absence of any commercial or financial relationships that could be construed as a potential conflict of interest.
